# IncHI2 Plasmid Encoding *bla*_CTX-M-55_ and *mcr-1.1* in Salmonella enterica SE20-C72-2 and Escherichia coli EC20-C72-1 Isolates from the Edible River Fish Anabas testudineus

**DOI:** 10.1128/mra.00149-23

**Published:** 2023-06-27

**Authors:** Tatsuya Nakayama, Shiori Yamamoto, Natsuki Ohata, Takahiro Yamaguchi, Michio Jinnai, Doan Tran Nguyen Minh, Oanh Nguyen Hoang, Hien Le Thi, Phong Ngo Thanh, Phuong Hoang Hoai, Phuc Nguyen Do, Chinh Dang Van, Yuko Kumeda, Atsushi Hase

**Affiliations:** a Graduate School of Integrated Sciences for Life, Hiroshima University, Hiroshima, Japan; b Division of Biomedical Food Research, National Institute of Health Sciences, Kawasaki, Kanagawa, Japan; c Department of Nutrition and Dietetics, Kamakura Women’s University, Kanagawa, Japan; d Department of Microbiology, Osaka Institute of Public Health, Osaka, Japan; e Department of Microbiology, Kanagawa Prefectural Institute of Public Health, Kanagawa, Japan; f Institute of Public Health, Ho Chi Minh City, Vietnam; g Research Center of Microorganism Control, Osaka Metropolitan University, Osaka, Japan; h Faculty of Contemporary Human Life Science, Tezukayama University, Nara, Japan; University of Delaware College of Engineering

## Abstract

Salmonella enterica SE20-C72-2 and Escherichia coli EC20-C72-1 were isolated from the edible fish Anabas testudineus in Vietnam. The chromosomes and plasmids from both strains were sequenced using Oxford Nanopore and Illumina sequencing. Plasmids approximately 250 kbp long, encoding *bla*_CTX-M-55_ and *mcr-1.1*, were detected in both strains.

## ANNOUNCEMENT

As antibiotic-resistant bacteria (ARB) have reportedly been found in imported foods, the spread of plasmid-mediated ARB through food products should be of concern (1). Here, we report the whole-genome sequence (WGS) of Salmonella enterica and Escherichia coli harboring a common plasmid isolated from edible river fish, Anabas testudineus, purchased in Ho Chi Minh City, Vietnam.

Fresh *Anabas testudineus* was purchased from a retail market in Ho Chi Minh City. The gut content of fish (5 g) was mixed with buffered peptone water (the composition [g/liter] included peptone [10.0], sodium chloride [5.0], potassium dihydrogen phosphate [1.5], and di-sodium hydrogen phosphate dodecahydrate [9.0]) (Merck, Darmstadt, Germany) (45 mL). After incubation at 37°C for 24 h, 100 μL of the culture was spread onto CHROMagar Salmonella and ECC agar (CHROMagar, Paris, France) containing cefotaxime (2 μg/mL) and incubated at 37°C for 24 h. Several mauve and blue, round colonies were selected and isolated as Salmonella and E. coli, respectively, and were then investigated for antibiotic susceptibility (2). After a subculturing step in LB medium at 37°C for 18 h, DNA was extracted using the DNeasy blood and tissue kit (Qiagen, Hilden, Germany) and Genomic-tip 100/G (Qiagen). The extracted DNA was checked using a Qubit double-stranded DNA (dsDNA) high-sensitivity (HS) assay (Thermo Fisher, Waltham, USA). For short- and long-read sequencing library preparation, a QIAseq FX DNA library UDI kit (Qiagen) and a rapid barcoding kit (Oxford Nanopore Technologies, Oxford, UK) were used, and sequencing was performed using a HiSeq instrument (Illumina, San Diego, USA) with a 2 × 150-bp paired-end protocol and MinION device (Oxford) with flow cell R9.4.1 (Oxford). After short-read sequences were obtained, trimming and quality checks were conducted using fastp v0.20.0 (3). Short- and long-read quality checks were performed with fastqc v0.11.9 (3) and Nanofilt v2.8.0 (4). Guppy v5.0.11 was used as the base caller. A hybrid assembly of Illumina (for Salmonella and E. coli total reads, 7,007,886 and 5,754,540; mean length after filtering, 2 × 147-bp reads [paired end]; total bases, 1,051.2 and 863.2 Mb) and MinION (total reads, 95,545 and 128,679; *N*_50_ value, 16,898 and 15,094 bp; total bases, 864.7 and 1,134.1 Mb) sequencing data was conducted using Unicycler v0.5.0 (5). Default parameters were used except where otherwise noted. Annotation was performed using DFAST software. The assembled WGS was analyzed with MLST2.0 and MobileElementFinder. SpeciesFinder confirmed that Salmonella and E. coli were isolated, and ResFinder analysis showed that *bla*_CTX-M-55_, *mcr-1.1*, *aac(3′)-lld*, *ARR-2*, *dfrA14*, *mph(A)*, and *qnrS1* were found in both plasmids (Table 1).

**TABLE 1:** Genome information of Salmonella SE20-C72-2 and E. coli EC20-C72-1 isolates

Characteristic	Data for:			
	Salmonella enterica SE20-C72-2	Plasmid pSE20-C72-2-1	Escherichia coli EC20-C72-1	Plasmid pEC20-C72-1-1
MLST·serotype/IncType	ST322·Schwarzengrund	IncHI2	ST206·H5	IncHI2
Total length (bp)	5,146,518	256,294	4,942,769	254,266
No of contigs	13	1	4	1
GC content (%)	51.80	46.00	50.70	46.70
*N*_50_ (bp)	4,720,010	256,294	4,599,111	254,266
No of CDSs^*a*^	4,889	274	4,664	274
No. of rRNAs	22	0	22	0
No. of tRNAs	83	0	88	0
Antibiotic resistance genes	*aac(6')-IB-cr*, *aadA2*, *aadA16*, *aadA1*, *aac(3)-IId*, *aac(3')-IV*, *aac(6')-Iaa*, *aph(4)-Ia*, *ARR-2*, *ARR-3*, *bla*_CTX-M-55_, *bla*_TEM_(-1B, 30, 104, 176, 198, 217, 230, 270, and 217), *cmlA1*, *dfrA12*, *dfrA14*, *dfrA27*, *floR*, *sul1*, *sul3*, *tet(A)*, *qacE*, *qacL*, *qnrS1*, *mcr-1.1*, *mph(A)*	*aac(3)-IId*, *ARR-2*, *bla*_CTX-M-55_, *blaTEM(-1B*, *30*, *104*, *176*, *198*, *217*, *230*, *270*, *and 217)*, *dfrA14*, *mcr-1.1*, *mph(A)*, *qnrS1*	*aph(3')-Ia*, *aac(3)-Iid*, *ARR-2*, *aac(3)-Iid*, *bla*_TEM-1B_, *mcr-1.1*, *bla*_CTX-M-55_, *qnrS1*, *mph(A)*, *dfrA14*, *qnrS1*, *tet(A)*, *floR*, *sul2*	*aph(3')-Ia*, *aac(3)-Ild*, *ARR-2*, *bla*_CTX-M-55_, *dfrA14*, *floR*, *mcr-1.1*, *mph(A)*, *qnrS1*, *tet(A)*
Accession no.	AP026948, AP026949, AP026950, AP026951, AP026952, AP026953, AP026954, AP026955, AP026956, AP026957, AP026958, AP026959, AP026960	AP026949	AP026961, AP026962, AP026963, AP026964	AP026962

a CDSs, coding DNA sequences.

**FIG 1:**
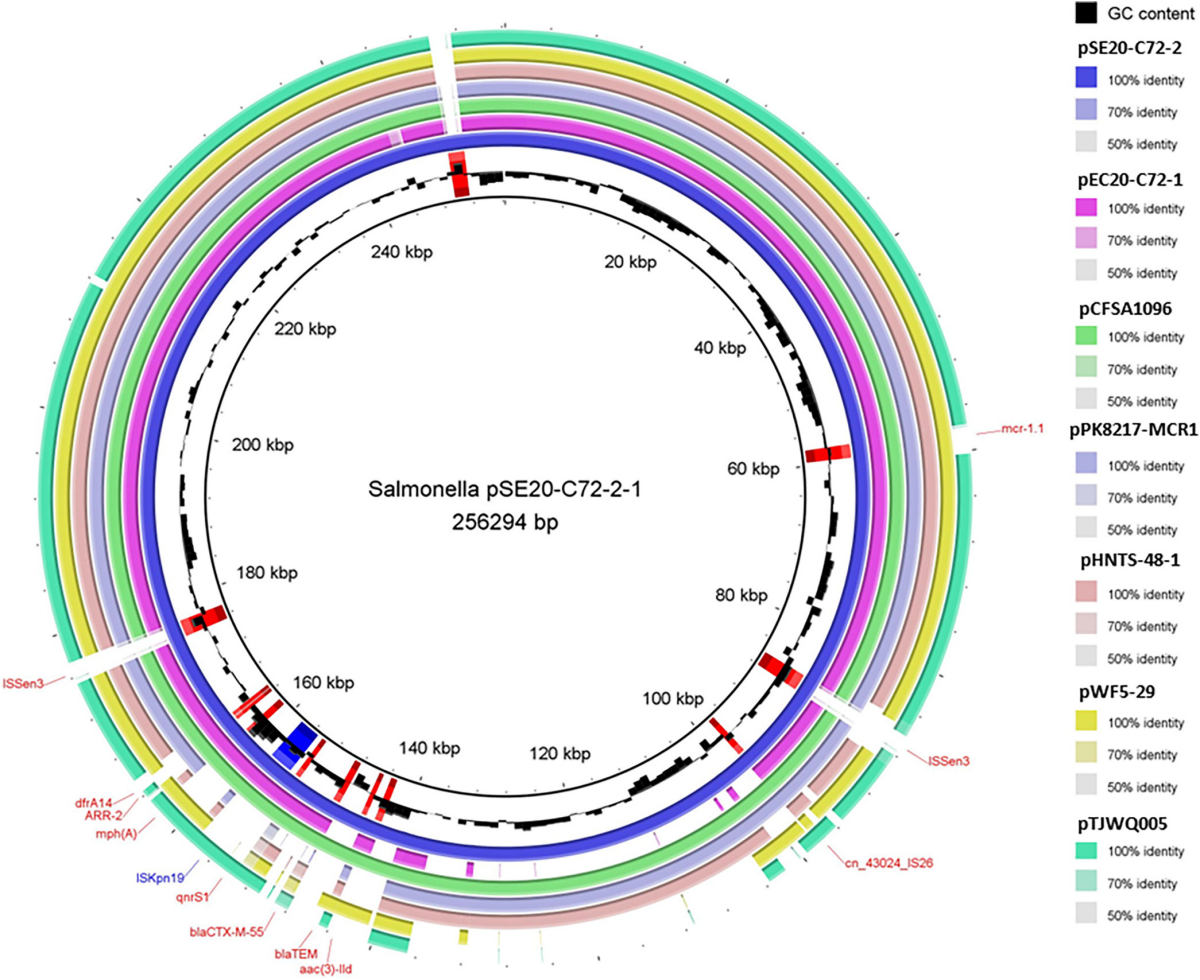
Complete plasmid map of pSE20-C72-2-1 from Salmonella enterica SE20-C72-2. The complete plasmids of six strains (GenBank accession no. AP026962) (pEC20-C72-1 from E. coli), CP033347 (pCFSA1096 from Salmonella), CP080122 (pPK8217-MCR1 from E. coli), MF135534 (pHNTS-48-1 from Raoultella ornithinolytica), MG385063 (pWF5-29 from E. coli), and CP040457 (pTJWQ005 from Salmonella) with high homology were selected. The reference strain was AP026949 (pSE20-C72-2-1 from Salmonella enterica SE20-C72-2). Plasmid maps were designed using BLAST ring image generator v0.95.

Comparisons by basic local alignment search tool (BLAST) and BLAST ring image generator showed that the plasmids carried by both bacteria are remarkably similar. The mobile genetic factors IS*26* and IS*Kpn19* are located upstream and downstream of *bla*_CTX-M-55_, *qnrS1*, and *aac(3′)-lld* (Fig. 1). Therefore, this shared plasmid could be horizontally transmitted.

### Data availability.

Salmonella SE20-C72-2 and E. coli EC20-C72-1 WGS as well as pSE20-C72-2-1 and pEC20-C72-1-1 plasmid genome sequencing results were deposited in DDBJ/GenBank (accession numbers AP026948, AP026949, AP026961, and AP026962). The raw reads were deposited under the accession numbers DRX382165, DRX382166, DRX382163, and DRX382164 with the BioProject number PRJDB11927.
